# Identification and Characterization of Novel Rat Polyomavirus 2 in a Colony of X-SCID Rats by P-PIT assay

**DOI:** 10.1128/mSphere.00334-16

**Published:** 2016-12-21

**Authors:** Lora H. Rigatti, Tuna Toptan, Joseph T. Newsome, Patrick S. Moore, Yuan Chang

**Affiliations:** a Division of Laboratory Animal Resources, University of Pittsburgh School of Medicine, Pittsburgh, Pennsylvania, USA; b Cancer Virology Program, University of Pittsburgh Cancer Institute, Pittsburgh, Pennsylvania, USA; University of Michigan

**Keywords:** epitheliotropism, immune suppression, KIV, P-PIT, WUV, X-SCID, inclusion body, laboratory animals, polyomavirus, simian virus 40

## Abstract

Polyomaviruses (PyVs) are known to infect a wide range of vertebrates and invertebrates and are associated with a broad spectrum of diseases, including cancers, particularly in immune-suppressed hosts. A novel polyomavirus, designated rat polyomavirus 2 (RatPyV2), was identified from a breeding colony of rats having X-linked severe combined immunodeficiency. Using a human panpolyomavirus immunohistochemistry test (P-PIT), RatPyV2 was initially detected in the parotid salivary gland of a colony member. Rolling circle amplification using DNA from harderian and parotid glands identified a novel 5.1-kb polyomavirus genome closely related to human Washington University (WU) and Karolinska Institute (KI) and vole polyomaviruses but notably divergent from *Rattus norvegicus* PyV1 (RnorPyV1; also designated RatPyV1). Further screening showed RatPyV2 inclusion body infection in the lung epithelium and variably in other respiratory, reproductive, and glandular tissues of 12/12 (100%) rats.

**IMPORTANCE** Although P-PIT was developed to detect diseases associated with known human polyomaviruses, the identification of a new polyomavirus in rats suggests that it may have utility as a broad-based screen for new, as well as known polyomaviruses. Our findings suggest that RatPyV2 may be a commensal infection of laboratory rats that can lead to disseminated disease in T cell immune-deficient rats. Infection of the X-SCID rats with RatPyV2 and *Pneumocystis carinii* is a potential model for coinfection pathogenesis and treatment options during transplant preclinical studies.

## Abstract (precis)

Although P-PIT was developed to detect diseases associated with known human polyomaviruses, the identification of a new polyomavirus in rats suggests that it may have utility as a broad-based screen for new, as well as known polyomaviruses. Our findings suggest that RatPyV2 may be a commensal infection of laboratory rats that can lead to disseminated disease in T cell immune-deficient rats. Infection of the X-SCID rats with RatPyV2 and *Pneumocystis carinii* is a potential model for coinfection pathogenesis and treatment options during transplant preclinical studies.

## INTRODUCTION

Polyomaviruses (PyVs) are a family of small DNA viruses known to infect a variety of mammals, birds, fish, and scorpions (1, 2). They are members of the family *Polyomaviridae* and are nonenveloped icosahedral viruses, with circular double-stranded DNA genomes of approximately 5,000 base pairs (bp) (3). An early viral gene region encodes multiple regulatory proteins, called tumor antigens (T-Ag), by alternative splicing, and a late gene region encodes structural capsid proteins, including VP1, VP2, and VP3 (4).

Most mammalian polyomaviruses cause subclinical infections with lifelong persistence in their natural hosts. According to serological studies, asymptomatic infection occurs with 12 of the 13 known polyomaviruses detected in humans (5–14). Polyomavirus-related diseases, which include nephritis (BK virus [BKV]) (15), encephalitis (JC virus [JCV]) (16), Merkel cell carcinoma (Merkel cell polyomavirus [MCV]) (17), skin dysplasia (trichodysplasia spinulosa-associated polyomavirus [TSV] and human polyomavirus 7 [HPyV7]) (18, 19), and pneumonitis (Washington University [WU] virus [WUV]) (20, 21), can occur among immune-suppressed individuals, including posttransplantation and AIDS patients. A panpolyomavirus immunohistochemistry test (P-PIT), comprised of three antibodies (Pab416, Xt7, and 2t2), recognizes well-conserved antigenic epitopes of polyomavirus early proteins and has been show to detect T antigens of all 13 human polyomaviruses (21). Toptan et al. found positive P-PIT staining for all currently known polyomavirus-related diseased human tissues.

To date, only seven distinct rodent PyVs have been fully sequenced: mouse PyV, mouse pneumotropic PyV, hamster PyV, *Mastomys* PyV, bank vole PyV, common vole PyV, and *Rattus norvegicus* PyV1 (RnorPyV1) (22–27). These polyomaviruses were largely found as commensal infections. RnorPyV1, closely related to mouse and hamster PyV, appears to persist with no signs of disease in feral Norway rats. However, in 1984, Ward et al. reported a widespread infection in a colony of athymic nude rats manifesting with parotid sialoadenitis, bronchitis, rhinitis, and harderian adenitis (28). Immunohistochemical (IHC) staining at the time with an anti-simian virus 40 (SV40) T antigen antibody was reactive with infected tissues; however, no viral sequences were obtained.

Here, we describe the identification of a new polyomavirus (RatPyV2) associated with disseminated viral inclusion body disease in X-linked severe combined immune deficiency (X-SCID) rats that have a genetically disrupted interleukin-2 receptor gamma gene (*IL2RG*) (29). The majority of these laboratory rats infected with RatPyV2 had decreased fecundity but were otherwise asymptomatic. However, some rats developed acute respiratory distress associated with *Pneumocystis carinii* and RatPyV2 coinfection, as well as chromodacryorrhea (red tear secretion from the harderian gland). Phylogenetic analyses based on large T (LT) sequences show that RatPyV2 belongs to the betapolyomavirus genus as proposed by the International Committee in Taxonomy of Viruses (ICTV) (30). Phylogenetic analysis with virus protein 1 (VP1), similar to LT analysis, shows RatPyV2 to be most closely related to human WU and Karolinska Institute (KI) polyomaviruses and more remotely related to RatPyV1.

## RESULTS

### Viral outbreak in X-SCID rat colony.

**FIG 1:**
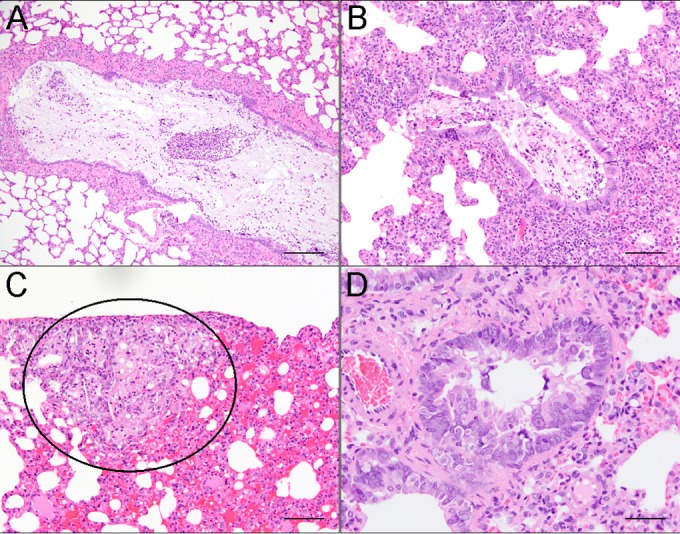
Lesions in hematoxylin-and-eosin (H&E)-stained lung sections of X-SCID rats infected with *Pneumocystis carinii*. (A) Bronchi were filled with neutrophils, mucous, foamy macrophages, and cell debris. Scale bar = 200 μm. (B) Bronchioles were filled with material similar to that in bronchi. Interstitial pneumonia was present in adjacent alveoli. Scale bar = 100 μm. (C) Subpleural foci consisted of alveoli filled with foamy macrophages and occasional cholesterol clefts (circled area), in addition to alveolar hemorrhage. Scale bar = 100 μm. (D) Bronchiolar hyperplasia was present, with intranuclear inclusion bodies within the bronchiolar epithelium. Scale bar = 50 μm.

During quarterly diagnostic screening, serologic positivity for *P. carinii* was detected in an X-SCID rat breeding colony. Four (2 male and 2 female) rats exhibiting respiratory distress and chromodacryorrhea were euthanized, and lungs were collected for histopathology evaluation (Fig. 1; see Fig. S1 in the supplemental material). PCR testing confirmed infection with *P. carinii* (data not shown), and all rats in the colony began treatment with 250 mg/kg of body weight/day sulfamethoxazole (SMZ) pulse treatment for 2 weeks orally in water bottles. Rats were given 2 weeks off and a subsequent second round of SMZ. Follow-up serology performed at quarterly testing indicated no active infection for *P. carinii* or for other known rat pathogens, including cytomegalovirus and mouse adenoviruses 1 and 2 (Table 1).

10.1128/mSphere.00334-16.1 Figure S1  Silver staining of *Pneumocystis carinii*-coinfected lung samples in areas of interstitial pneumonia in H&E-stained tissue sections (left, top right) revealed black staining of cyst walls (bottom right). Left, ×20 magnification; right, ×100 magnification. Download Figure S1, PDF file, 0.9 MB. Copyright © 2016 Rigatti et al. 2016 Rigatti et al. This content is distributed under the terms of the Creative Commons Attribution 4.0 International license.

**TABLE 1:** Pathogens included in quarterly (3 months) rodent surveillance testing

Test type	Pathogen
Serology	Sendai virus (SEND)
	Pneumonia virus of mice (PVM)
	Sialodacryoadenitis virus (SDAV)
	Kilham rat virus (KRV, RV)
	Toolan’s H1 virus (H-1)
	Rat parvovirus (RPV)
	Rat minute virus (RMV)
	Reovirus (REO-3)
	Rat theilovirus (RTV, TMEV, REV)
	Rat coronavirus (RCV)
	*Pneumocystis carinii* (previously thought to be rat respiratory virus [RRV])
	Lymphocytic choriomeningitis (LCMV)
	Hantaan virus (HANT)
	Mouse adenovirus (MAV)
	Cilia-associated respiratory bacillus (CARB)
	*Mycoplasma pulmonis* (MPUL)
	*Encephalitozoon cuniculi* (ECUN)
PCR	Pinworms of the genera *Aspiculuris* and *Syphacia*
	Ectoparasites

Other than the 4 rats mentioned above, no rats within the colony displayed overt clinical symptoms. However, from a reproductive standpoint, female rats within this colony usually had 4 to 8 pups per litter, and the reproductive life span consisted of 2 litters per animal. Rarely, females had third litters with very poor survival rates, suggesting markedly reduced fecundity. Female rats were unable to maintain pregnancy after 6 months of age.

**FIG 2:**
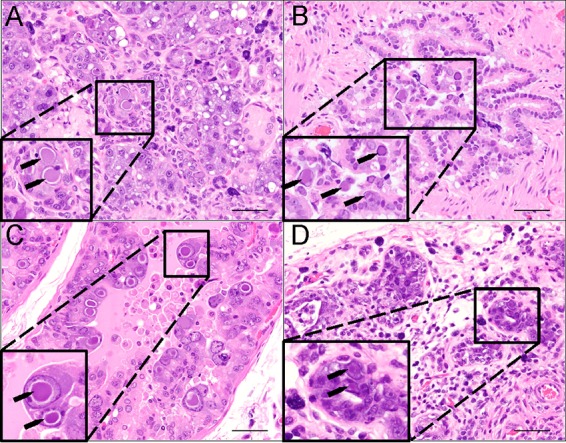
Hematoxylin-and-eosin (H&E) staining of multiple organs in X-SCID rats reveals epithelial hyperplasia with cytomegaly and intranuclear inclusion bodies (black arrows). (A) Parotid salivary gland acinar epithelium was hyperplastic, with regions of necrosis and loss. The interstitium was expanded by lymphocytes, plasma cells, and mast cells. (B) Bronchiolar epithelium was hyperplastic, with enlarged apical epithelial cells containing intranuclear inclusions. (C) Prostate glandular epithelium was severely hyperplastic and disorganized. Cytomegalic cells with extremely large intranuclear inclusions were a prominent feature. (D) There was severe loss of glands within the harderian gland. The remaining glands were often hyperplastic and disorganized, with areas of necrosis. Regions between the remaining glands contain fibrous connective tissue, lymphocytes, plasma cells, and mast cells. Scale bars = 50 μm.

Although diagnostic screening excluded all known rat pathogens except *P. carinii*, histopathological changes, such as inclusion bodies, suggested a viral cause that could not be explained by *P. carinii* infection of the lung alone (Fig. 1). Therefore, a total of 8 additional rats (6 adults and 2 6-week-old weanlings) were examined via gross and microscopic pathology of all organs to look for pathological and immunohistochemical evidence of viral disease. Characteristic gross findings expected for the X-SCID strain (29) included severe thymic hypoplasia, unidentifiable lymph nodes, and hypoplastic spleens. In addition to gross findings indicative of pneumonia (see Fig. S2 and Table S2 in the supplemental material), we observed microscopic alterations, including intranuclear inclusions (Fig. 2), inflammation, and hyperplastic and dysplastic changes in the epithelia of multiple organs: nasal mucosa and lung (Fig. 1), parotid and submandibular salivary and harderian glands, reproductive organs (prostate and uterine epithelium), and kidney (see Table S3 in the supplemental material).

10.1128/mSphere.00334-16.2 Figure S2  (A) Gross lung lesions were present in polyomavirus-infected rats. Lungs were mottled red-pink with 1- to 4-mm white pleural foci. Scattered similarly sized red foci were present on the pleural surface and throughout the lungs. Cranioventral atelectasis (arrow) was also evident within the cranial lobe of the right lung. (B) Control, normal rat lungs. Download Figure S2, PDF file, 0.8 MB. Copyright © 2016 Rigatti et al. 2016 Rigatti et al. This content is distributed under the terms of the Creative Commons Attribution 4.0 International license.

In the 1984 report mentioned above, a similar condition associated with polyomavirus infection was described in athymic nude rats (28). We therefore applied a recently developed panpolyomavirus immunohistochemistry test (P-PIT) comprised of a cocktail of three antibodies that, when combined, can detect all known human PyV T antigens (21).

**FIG 3:**
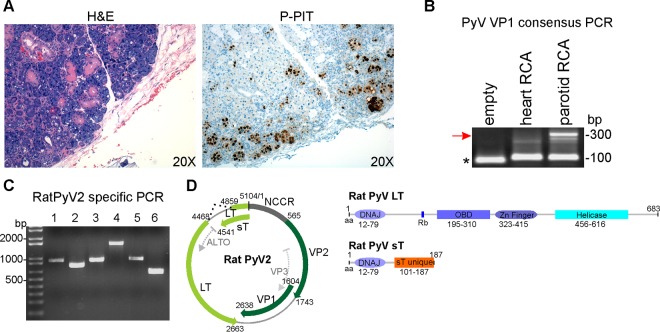
(A) Left, P-PIT staining of parotid gland. Right, H&E staining of the parotid salivary gland shows multiple viral inclusion bodies. Immunostaining with P-PIT antibody cocktail PAb416, xt7, and 2t2 was positive (brown) largely in the nucleus of infected cells. (B) Consensus PCR for PyV VP1. Rolling circle-amplified (RCA) DNA from FFPE heart and parotid tissues was subjected to nested PCR using consensus primers. Agarose gel electrophoresis shows an ~300-bp band (red arrow). Primer dimers are indicated by the asterisk. (C) Whole-genome amplification by RatPyV2-specific primers. Virus-specific primers (see Table S1 in the supplemental material) were designed and used to generate 6 overlapping PCR products. Lanes contain products amplified with the indicated primer pair: 1, F2-R3; 2, F4-R5; 3, F6-R7; 4, F8-R10; 5, F11-R12; 6, F13-R1. (D) Genome map of RatPyV2. The RatPyV2 genome comprises 5,104 base pairs (bp). The putative early region encodes large T (LT) and small T (sT) antigens (both light green). Intron splice sites for LT are indicated with dotted black lines. The putative late region comprises viral capsid proteins (VP1 and VP2; both dark green). The noncoding control region (NCCR; dark grey) contains the putative origin of replication and promoters. Examples of predicted open reading frames (ORFs) (ALTO-like ORF and VP3) are indicated by dotted light grey arrows in the early and late region, respectively. Schematic diagram on the right shows putative conserved domains of LT and sT. Amino acid (aa) positions for conserved DNAJ, pocket protein binding (Rb), origin binding (OBD), helicase, and unique sT domains are shown.

In all 12 rats, tissue sections showing intranuclear inclusion bodies by hematoxylin-and-eosin (H&E) examination also demonstrated correspondingly strong nuclear immunoreactivity by P-PIT (data not shown; representative examples are presented in Fig. 3A and 7). Tissues not affected by inclusion body changes, such as the heart, liver, spleen, and brain, were immunonegative (data not shown). Some degree of selectivity or tropism for distinct types of epithelia is discernible. For example, lesions in the salivary gland (Fig. 3A) were significantly more prominent in the serous portions of the gland than in the mucinous portions, where there were rare areas of necrosis, mixed infiltrates, and intranuclear inclusions. Follow-up immunostaining using each of the three antibodies comprising the P-PIT cocktail singly revealed strong PAb416 reactivity and weak Xt7 reactivity, while 2t2 antibody was unreactive on the diseased tissues (data not shown).

10.1128/mSphere.00334-16.4 Table S1  Primers used in this study. Download Table S1, PDF file, 0.1 MB. Copyright © 2016 Rigatti et al. 2016 Rigatti et al. This content is distributed under the terms of the Creative Commons Attribution 4.0 International license.

10.1128/mSphere.00334-16.5 Table S2  Gross findings present in RatPyV2-infected rats. N/A, not applicable because organ was not examined in this group. Download Table S2, PDF file, 0.1 MB. Copyright © 2016 Rigatti et al. 2016 Rigatti et al. This content is distributed under the terms of the Creative Commons Attribution 4.0 International license.

10.1128/mSphere.00334-16.6 Table S3  Histopathologic lesions in RatPyV2-infected rats. *, interstitial pneumonia was characterized by mixed interstitial inflammatory infiltrates and type II pneumocyte hyperplasia; **, intranuclear inclusion bodies were present in epithelial cells of these organs in every group where tissue was examined; ***, lesion was only present in a single male examined; N/A, not applicable because organ was not examined in this group; —, lesion not present. Download Table S3, PDF file, 0.1 MB. Copyright © 2016 Rigatti et al. 2016 Rigatti et al. This content is distributed under the terms of the Creative Commons Attribution 4.0 International license.

### Genome characterization and phylogenetic analysis of RatPyV2.

Rolling circle amplification (RCA) products obtained using the DNA extracted from formalin-fixed, paraffin-embedded (FFPE) sections of parotid salivary gland and heart tissues were amplified by polyomavirus consensus PCR for conserved LT (data not shown) and VP1 sequences (Fig. 3B). In concert with the IHC staining, consensus PCR using degenerate primers and parotid tissue DNA resulted in ~300-bp products for VP1 and T-Ag. Based on the initial sequencing analysis, we generated virus-specific primers for genome walking (see Table S1 in the supplemental material). We obtained six overlapping PCR products using fresh tissue DNA isolated from the harderian and parotid glands of an infected rat (Fig. 3C) which were sequenced to 2×depth. The sequences from both tissues were identical. Assembly of overlapping PCR segments revealed a previously unknown polyomavirus, which we designated RatPyV2. The RatPyV2 genome (GenBank accession number KX574453) is 5,104 bp in length and has gene syntheny with other polyomaviruses. The genome encodes open reading frames for 683-amino-acid (aa) large T (LT), 187-aa small T (sT), 34- aa VP1, and 393-aa VP2 proteins (Fig. 3D). RatPyV2 LT splicing is predicted to occur, with a splice donor site between nucleotides 4853 and 4862 (CAAG/GTACAT) and an acceptor site between nucleotides 4467 and 4480 (TTTTTTTTAAAG/GT) within the large T sequence, which would generate a predicted protein of 683 aa (Fig. 3D). Motif analysis shows the putative LT protein to possess conserved PyV domains, including a DnaJ domain (aa 12 to 79, HPDKGG box), an Rb-binding site (aa 160 to 164, LHCDE), an origin binding domain (aa 195 to 310), a zinc finger domain (aa 323 to 415), and a helicase domain (aa 456 to 616) (Fig. 3D). The unspliced early region transcript is also predicted to encode an sT protein with conserved protein phosphatase 2A binding site residues. Overall, RatPyV2 shares 49.13% genome identity with RnorPyV1 (accession number KR075946.1).

**FIG 4:**
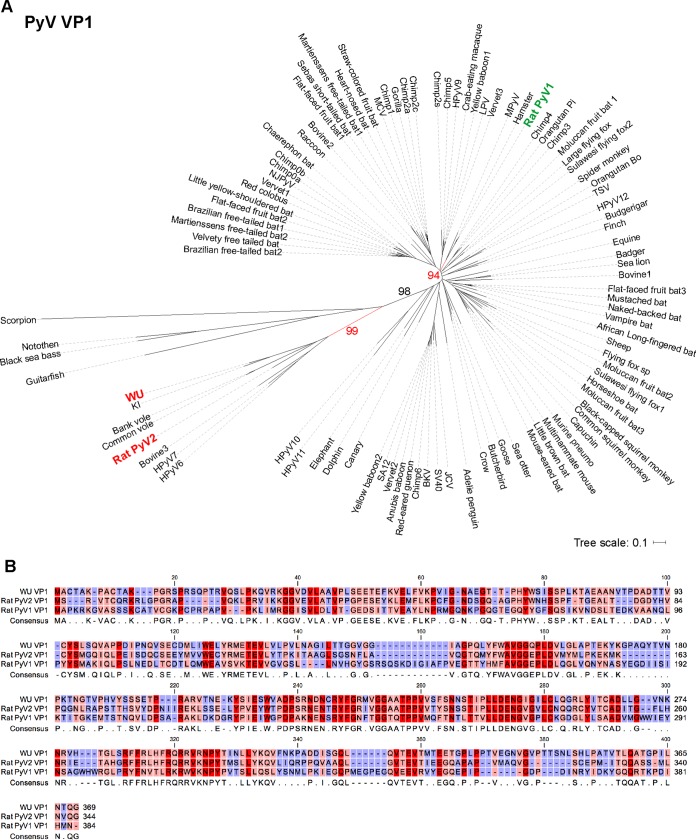
Phylogenetic analysis of 100 full-length PyV VP1 protein sequences. (A) Consensus tree of 1,000 bootstrap replicates was generated by the neighbor-joining method using MEGA7. Percent bootstrap values are given for selected nodes. RatPyV2 (red, boldface) is closely related to WU (red, boldface) and highly divergent from RatPyV1 (green, boldface). The phylogenetic tree was visualized using iTOL. (B) VP1 amino acid sequence alignment of WU, RatPyV1 (RnorPyV1), and RatPyV2 sequences was generated using CLC Genomics Workbench software. Residue conservation ranges from red (conserved) to pink (mostly conserved) to blue (divergent). A minus sign indicates missing residue at the given position.

**FIG 5:**
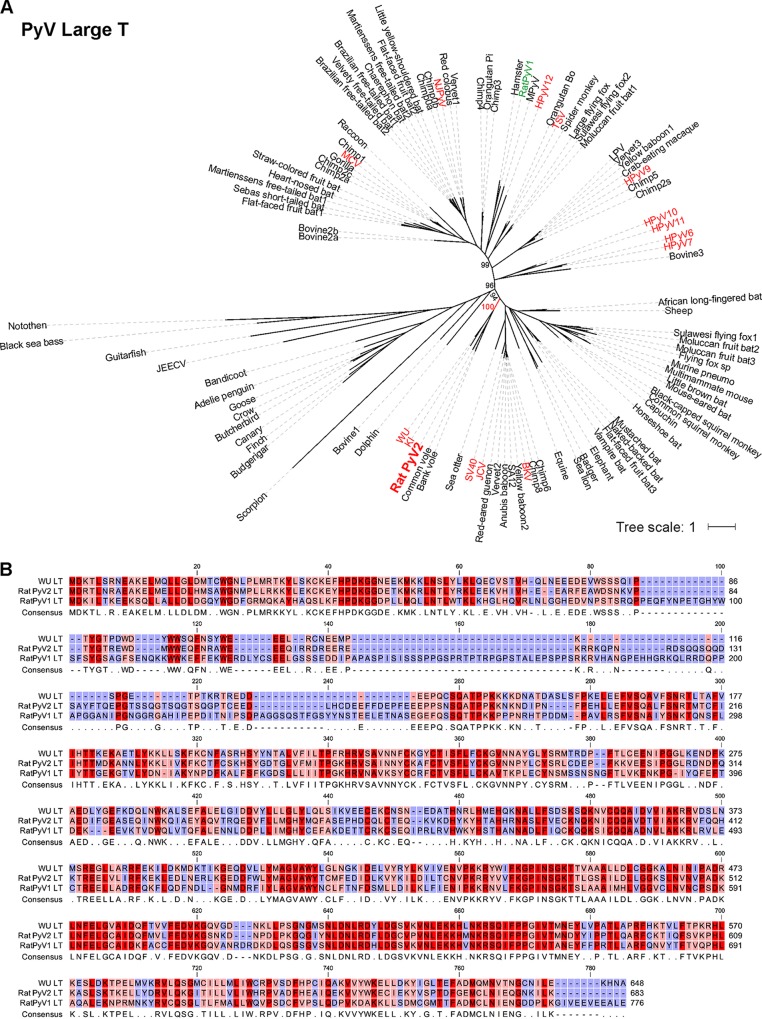
Phylogenetic analysis of 103 full-length PyV LT protein sequences. (A) Consensus tree of 1,000 bootstrap replicates was generated by the neighbor-joining method using MEGA7. Percent bootstrap values are given for selected nodes. All known P-PIT-reactive PyV T antigens are shown in red. RatPyV1 is shown in green. The phylogenetic tree was visualized using iTOL. (B) LT amino acid sequence alignment of WU, RatPyV1, and RatPyV2 sequences was generated using CLC Genomics Workbench software. Residue conservation ranges from red (conserved) to pink (mostly conserved) to blue (divergent). A minus sign indicates missing residue at the given position.

**FIG 6:**
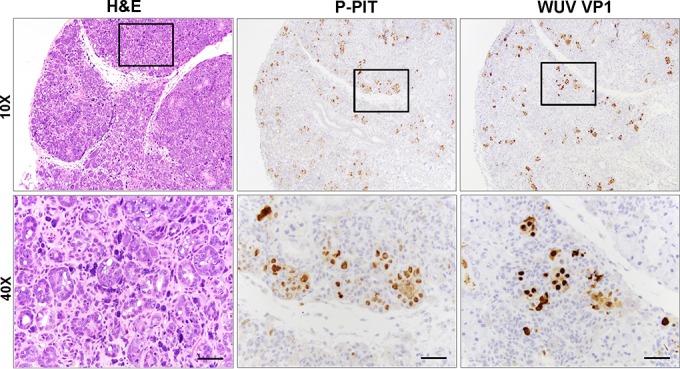
Immunohistochemistry analysis of parotid salivary gland tissue samples. H&E staining (left) showed glandular hyperplasia and inclusion bodies. P-PIT (middle) and WUV VP1 (right) staining correlated with H&E findings and revealed RatPyV2 T antigen and VP1 expression in these lesions. Scale bar = 100 μm. Images are shown at 10× and 40× the original magnification.

Phylogenetic analysis of VP1 and LT protein sequences revealed that RatPyV2 is most closely related to WUV, KIV, and vole PyVs (Fig. 4 and 5). Consistent with this close homology, IHC staining of parotid gland tissue with WUV VP1-specific antibody was strongly positive and correlated with PAb416 staining (Fig. 6).

### RatPyV2 is associated with disseminated inclusion body disease in X-SCID rats.

**FIG 7:**
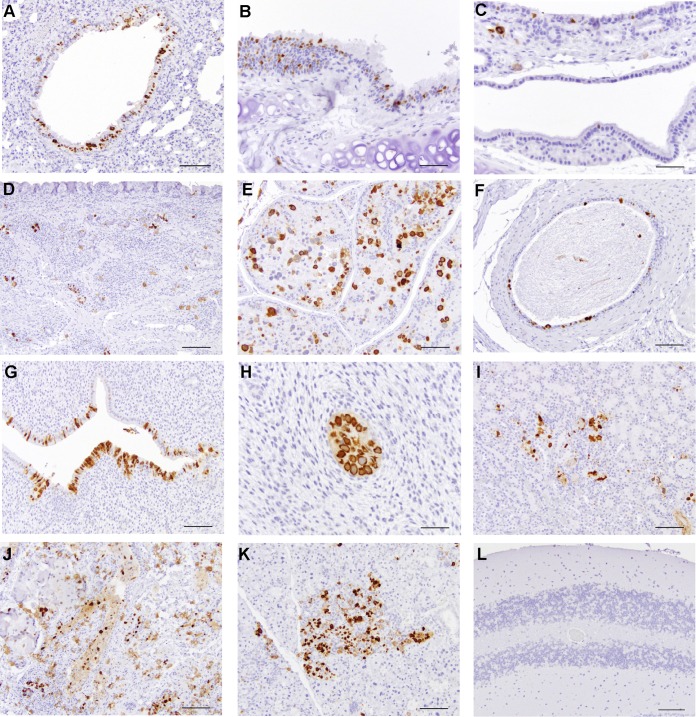
Representative images from immunohistochemistry performed on all organs of adult rats (female and male) with PAb416. Positively stained tissues included bronchiolar epithelium of the lung (A), tracheal epithelium and submucosa (B), laryngeal mucosa and submucosa (C), nasal mucosa and submucosa (D), prostate gland epithelium (E), vas deferens epithelium (F), uterine endometrial epithelium (G), uterine endometrial gland (H), focally damaged renal tubules within the kidney (I), harderian gland epithelium (J), salivary gland acini (K), and cerebellum (L). Scale bar = 100 µm (A, D, E, F, G, I, J, K, and L); scale bar = 50 µm (B, C, and H).

Immunohistochemistry was performed on organs of two adult rats with PAb416 antibody (Fig. 7). Positively staining tissues included bronchiolar epithelium of the lung, tracheal epithelium and submucosa, vas deferens epithelium, prostate gland epithelium, laryngeal mucosa and submucosa, nasal mucosa and submucosa, uterine endometrial epithelium, uterine endometrial glands, harderian gland epithelium, salivary gland acini, and focally damaged renal tubules in the kidney of one rat.

After the initial 12 animals were characterized, a female breeder was sacrificed postpartum for complications related to parturition (retained placenta and endometritis). In addition to the previously described respiratory and glandular lesions, prominent intranuclear inclusion bodies were present in the epithelium of active mammary glandular epithelium, and pAB416 staining was strongly positive in these areas (see Fig. S3 in the supplemental material). Although the pathogenesis of RatPyV2 is still unclear, transmission via milk (or colostrum) is a potential cause of spread to young animals within the colony.

10.1128/mSphere.00334-16.3 Figure S3  The intranuclear inclusion bodies present in the epithelium of active mammary glandular epithelium are strongly positive (brown) for pAB416 staining. Images are at 4× (left) and 20× (right) the original magnification. Download Figure S3, PDF file, 2.8 MB. Copyright © 2016 Rigatti et al. 2016 Rigatti et al. This content is distributed under the terms of the Creative Commons Attribution 4.0 International license.

**FIG 8:**
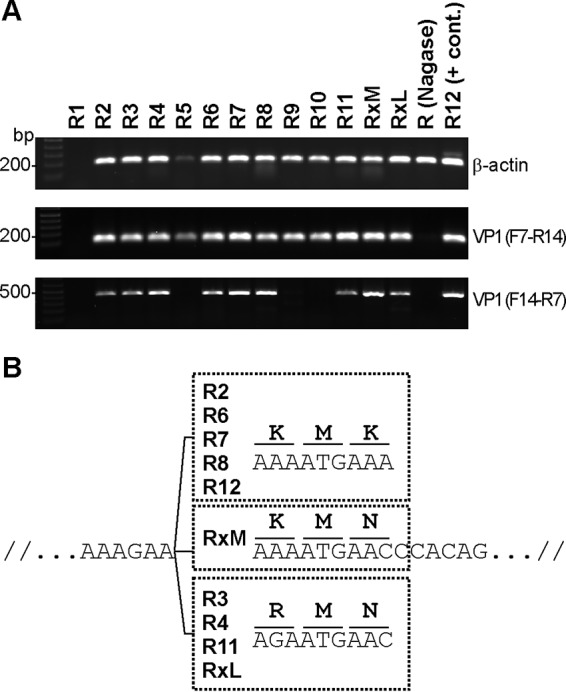
PCR detection of RatPyV2 infection in entire X-SCID rat colony. (A) DNA was extracted from FFPE lung tissue sections of all 12 X-SCID rats (R1 to R12), a female rat (Rx) with postpartum complications (mammary tissue, RxM, and lung tissue, RxL), and an immunocompetent Nagase strain rat [R (Nagase)]. β-Actin PCR products (191 bp) are amplifiable for all rat samples except those from R1. VP1 products (103 bp; primers F7-R14) were detected in all X-SCID rats (R2 to R12 and Rx) but not in the Nagase strain rat. A 432-bp fragment of VP1 was amplified using the F14-R7 primer pair and analyzed by sequencing. (B) Single nucleotide polymorphisms (SNP) detected in tissues from different rats are shown. For R3, R4, R11, and Rx (from both mammary and lung sites), an SNP at nucleotide position 2092 (A/C) leading to a K/N amino acid change was detected. R3, R4, R11, and RxL demonstrated an SNP at nucleotide position 2085 (A/G) leading to a K/R amino acid change.

We further verified that RatPyV2 infection was disseminated throughout the colony by PCR and sequencing analysis. Using DNA extracted from FFPE lung tissues from all 12 rats (R1 to R12) and the female rat (Rx) mentioned above that presented with postpartum complications, we performed PCR using primers for the β-actin housekeeping gene and RatPyV2 VP1 (Fig. 8A). R12, from which we sequenced the complete RatPyV2 genome, was used as the positive control. As a negative control, we used lung tissue DNA from an immunocompetent Nagase strain rat that shared the same cubicle with the X-SCID colony. We detected β-actin PCR products from all samples except those from R1, indicating suboptimal extracted DNA integrity for PCR studies. VP1 PCR products of 103 bp (amplified using primer pair F7-R14; see Table S1 in the supplemental material for primers) were observed in all β-actin-positive samples except those from the Nagase strain rat used as a negative control. For sequence confirmation and analysis, a larger region of VP1 was amplified using a different primer pair (primer pair F14-R7; see Table S1). Although the DNA from two samples (R9 and R10) was not amplifiable over this larger genomic region, sequencing of the remaining 10 samples, spanning nucleotides 1885 to 2316, revealed two single nucleotide polymorphisms leading to changes in amino acids at nucleotide positions 2085 (A/G) and 2092 (A/C) in different rats (Fig. 8B).

## DISCUSSION

In this study, we identified a novel rat polyomavirus infection in an X-SCID rat colony using the recently developed P-PIT assay that is designed to identify human polyomaviruses. This allowed us to isolate RatPyV2 sequences from immunoreactive tissues. Being immunocompromised, these rats are susceptible to a variety of opportunistic infections. The X-SCID rat colony was first suspected to harbor* P. carinii* as a cause for the respiratory symptoms. Following treatment of this colony for* P. carinii*, clinical symptoms subsided; however, evidence of viral disease was still present on histopathology despite negative tests for a comprehensive list of pathogens (Table 1), leading to identification of RatPyV2. *P. carinii* has long been known to cause pneumonia in immunosuppressed rats, but over the last several years, studies have also linked *P. carinii* to infectious interstitial pneumonia (IIP), a common, transient, usually mild disease of immunocompetent laboratory rats that had previously been attributed to an alleged virus referred to as rat respiratory virus (31, 32). Although RatPyV2 caused significant histopathologic lesions in multiple organs, including the lungs, clinical symptoms, such as respiratory distress and chromodacryorrhea, were only manifested by rats coinfected with *P. carinii*. However, in the majority of the colony, we observed reduced fecundity independent of *P. carinii* infection.

Ward et al. in 1984 described electron microscopic evidence for polyomavirus infection in 32 athymic nude (NIH-*rnu*) rats (28) that possessed spontaneous mutations in the *FOXN1* gene (33). The pathology described by these authors included inclusion bodies in the salivary glands, harderian glands, lungs, and nasal glands, findings that are similar those in our X-SCID (*IL2RG*-SCID) rats infected with RatPyV2. Both *FOXN1* and *IL2RG* mutations generate a T cell immunodeficient phenotype in experimental animals and in humans (29, 34–40). While it is not known whether RatPyV2 was responsible for the disease described in the NIH-*rnu* nude rats by Ward et al. (28), these findings are consistent with T cell surveillance being critical for host control of rat polyomavirus replication. The identification of this tractable virus-host model can allow experimental manipulation to assess the role of T cell immune surveillance in polyomavirus replication control.

In addition to IHC analysis performed on different tissues of the 12 rats, we further verified the extent of infection in this colony by PCR using RatPyV2-specific primers for regions in the VP1 locus. All 12 rats showed pulmonary infection by RatPyV2. However, the IHC and PCR assays used in this study do not address whether additional rat polyomaviruses are present.

Additional studies are needed to define the natural history of RatPyV2 infection. It is not known whether this is a common commensal infection of rats or if this is a rare and isolated infection. Based on the biology of other mammalian polyomaviruses, we suspect RatPyV2 to be a relatively common infection of rats that manifests as a disease in a setting of severe T cell immunodeficiency. If RatPyV2 is common, control measures might be devised (such as breeding RatPyV2-free rodents) to improve experimental animal health. Whether RatPyV2 causes disease in immunocompetent rats is also unknown, but our study reveals that this can be readily assayed with available antibodies and by PCR.

Polyomavirus-host evolutionary studies suggest coevolution of polyomaviruses with their hosts (41). Given the broad phylogenetic radiation of polyomaviruses among rodents, it is likely that additional rat polyomaviruses are present in *Rattus* populations. More importantly, both *FOXN1* and *IL2RG* mutations are responsible for genetic T cell immunodeficiency syndromes in children (34). If T-cell immune surveillance is particularly critical for commensal polyomavirus control, our findings highlight the need to search for unrecognized polyomavirus infections as causes for disease in susceptible populations. Although P-PIT was not developed to discover nonhuman polyomaviruses, its identification of this agent in rats suggests that it may have utility as a broad-based screen for new, as well as known human polyomaviruses.

## MATERIALS AND METHODS

### Animals.

X-SCID rats were obtained from Kyoto University, Institute of Laboratory Animals, Graduate School of Medicine, Kyoto, Japan, on 2 June 2015 subsequent to a 3-month quarantine at Charles River, Inc. (Wilmington, MA). They were housed in numbers of 2 adults or fewer per static microisolator cage on sterilized hardwood chip bedding, fed irradiated Purina LabDiet rodent chow 5P76, and given water *ad libitum*. All rats were housed in a separate cubicle within the animal facility’s repository. The breeding scheme of this colony was pair-timed mating with weaning at 28 days.

All animal procedures conformed to NIH guidelines for the care and use of laboratory animals and were approved by the Institutional Animal Care and Use Committee at the University of Pittsburgh (protocol number 16048182). The University of Pittsburgh’s Animal Care and Use Program (ACUP) (unit number 000496) is fully accredited by the Association for the Assessment and Accreditation of Laboratory Animal Care International (AAALAC).

### Testing for rat infections.

For rodent health surveillance and disease detection, quarterly diagnostic screening, including serology (multiplexed fluorometric immunoassay [MFIA]), PCR, and parasitology, were performed by Charles River, Inc., Research Animal Diagnostic Services (Wilmington, MA) (Table 1). At each bedding change, soiled bedding from each of the cages was added to the sentinel (Sprague-Dawley rat) cage, thus exposing the sentinels to diseases carried by colony animals. For lung sections, Grocott’s methenamine silver (GMS) staining was performed on 5-µm-thick tissue sections for the detection of fungal elements.

### Histology and immunohistochemical staining.

Tissues were fixed in 10% neutral buffered formalin, embedded in paraffin, sectioned at 5-µm thickness, and stained with hematoxylin and eosin for evaluation by light microscopy. Immunohistochemistry staining was performed according to a previously published protocol (21). Briefly, slides were deparaffinized in xylene and rehydrated through sequential ethanol solutions. Following incubation with 3% hydrogen peroxide for 15 min, epitope retrieval was performed using 1 mM EDTA buffer, pH 8.0 (125°C for 3 min and 90°C for 15 s), in an antigen retrieval chamber (Decloaking Chamber; Biocare Medical). Sections were incubated with serum-free protein block buffer (Dako), followed by primary antibodies diluted in antibody buffer (1% bovine serum albumin [BSA], 0.1% gelatin, 0.5% Triton X-100, 0.05% sodium azide in phosphate-buffered saline [PBS], pH 7.4) overnight at 4°C in a humidified chamber. The antibodies used include PAb416 (clone Ab-2, catalog number DP02, 1:200; Millipore), 2t2 (1:5 hybridoma supernatant, or approximately 8-μg/ml concentrated monoclonal antibody [MAb]), Xt7 (1:5 hybridoma supernatant, or approximately 8-μg/ml concentrated MAb), and mouse WUV VP1 MAb (NN-Ab06, 1:2,000; kind gift of David Wang [42]). Tissue sections were developed with mouse EnVision polymer (Dako) for 30 min at room temperature, reacted with diaminobenzidine (DAB; Dako), and counterstained with hematoxylin (Dako).

### Genomic DNA extraction from FFPE or fresh tissue.

DNA was extracted from formalin-fixed, paraffin-embedded (FFPE) sections of heart and parotid gland tissues using the QIamp DNA FFPE tissue kit according to the manufacturer’s instructions. For genomic DNA isolation from fresh tissues, harderian and parotid gland tissues were minced in 1 ml of PBS, transferred to microcentrifuge tubes, and centrifuged for 5 min at 6,000 × *g*. The tissue pellets were incubated in 0.5 ml of lysis buffer (10 mM Tris-HCl, pH 8.0, 100 mM NaCl, 25 mM EDTA, pH 8.0, 1% SDS) with proteinase K (Promega) added to a 0.5-mg/ml final concentration overnight at 56°C with gentle shaking. This tissue solution was extracted with an equal amount of phenol chloroform-isoamyl alcohol (25:24:1; Amresco) for 10 min at room temperature and centrifuged for 5 min at 16,000 × *g* (Eppendorf 5424R). The aqueous layer was transferred into a new tube and mixed with 0.5 vol of 7.5 M ammonium acetate, 2 vol of 100% ethanol. After centrifugation for 5 min at 16,000 × *g*, the DNA pellet was rinsed with 70% ethanol, air dried, and subsequently dissolved in Tris-EDTA (TE) buffer.

### Rolling circle amplification, PCR, and sequencing.

FFPE tissue DNA was subjected to rolling circle amplification (RCA) using phi29 DNA polymerase (TempliPhi; GE Healthcare). Consensus PCR was carried out using 1/10 of the RCA template, consensus primers (see Table S1 in the supplemental material), and 2.5 U of *Taq* polymerase (NEB) supplemented with Thermopol buffer. The cycling conditions for HPyV T-Ag primers or VP1-1 primers were initial denaturation for 5 min at 95°C, followed by 45 cycles each of 95°C for 30 s, 45°C for 1 min, and 68°C for 1 min and a final elongation at 68°C for 5 min. Nested PCR was performed with consensus VP1-2 primers, using 4 µl of the first PCR product as the template, at 95°C for 5 min, 45 cycles each of 95°C for 30 s, 53°C for 1 min, and 68°C for 30 s, and a final extension at 68°C for 5 min. PCR products were separated by agarose-gel electrophoresis, and DNA was purified using the QIAquick gel extraction kit (Qiagen) prior to sequencing (MCLAB). Based on the initial sequencing analysis, virus-specific contig primers (see Table S1 in the supplemental material) were synthesized (IDT) and used to amplify the entire genome with Q5 high-fidelity *Taq* polymerase (NEB). For genome-walking PCR, we used DNA isolated from fresh harderian and parotid gland tissues. The cycling conditions for overlapping contigs were 3 min at 98°C, followed by 35 cycles each of 98°C for 10 s, 60 to 66°C for 30 s, and 72°C for 1 min and a final elongation at 72°C for 2 min. PCR samples were separated on a 1% agarose gel, extracted (Qiagen), and sequenced (MCLAB). Sequence analysis and genome assembly were done using ContigExpress and Vector NTI software (Invitrogen). Analysis of the predicted protein sequences for family domains was performed with Motif Scan on the European Bioinformatics Institute (EBI) website (http://www.ebi.ac.uk/).

### Phylogenetic analysis.

Currently, known polyomavirus annotated maps and protein sequences are given at http://home.ccr.cancer.gov/Lco/PyVE.asp. The full-length VP1 and LT protein sequences were aligned by ClustalW, and the phylogenetic trees were generated by the neighbor-joining method with 1,000 bootstrap replicates, implemented in the Molecular Evolutionary Genetics Analysis version 7 (MEGA7) software package (43). The phylogenetic trees were visualized using iTOL (http://itol.embl.de/) (44).

### Accession number(s).

The RatPyV2 genome has been deposited in GenBank under accession number KX574453.
