# Supplementary figures and images for: Identification and Characterization of Novel Rat Polyomavirus 2 in a Colony of X-SCID Rats by P-PIT assay
Source: mSphere. 2016 Dec 21;1(6):e00334-16. doi: 10.1128/mSphere.00334-16 (PMC5177731; doi:10.1128/mSphere.00334-16)

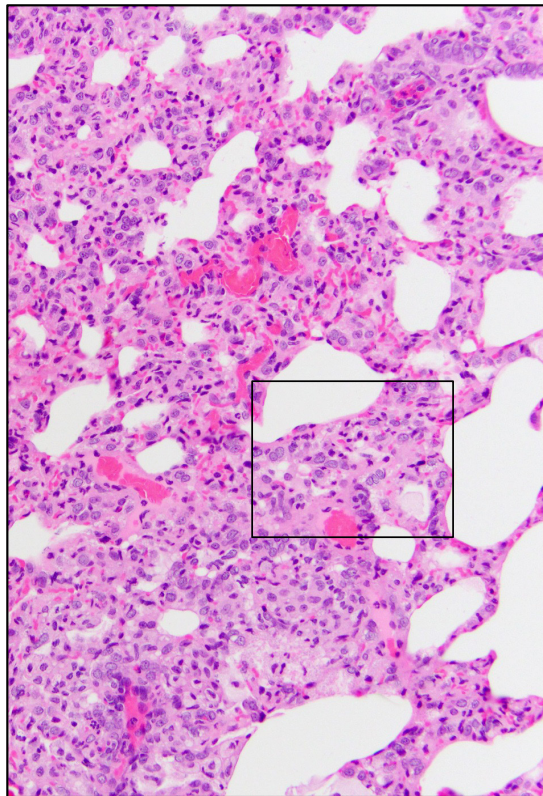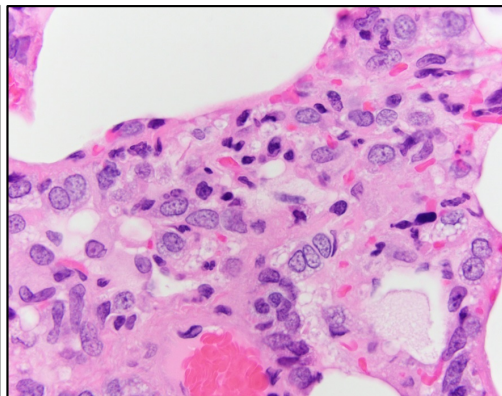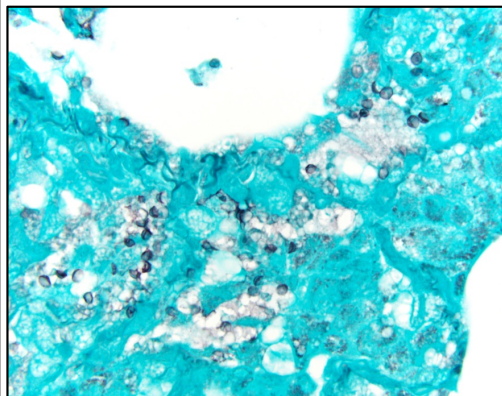

Supplement: Figure S1 [file sph006162212sf1.pdf]

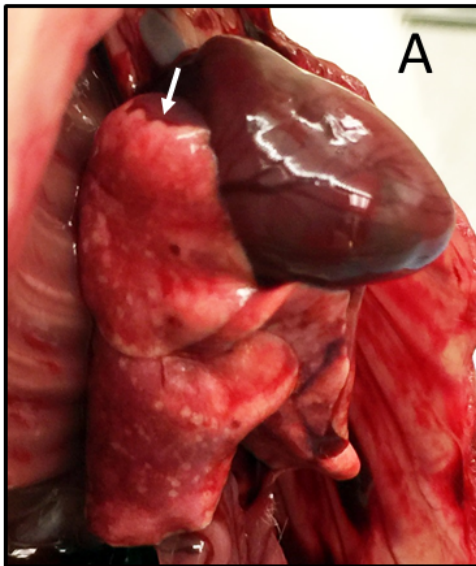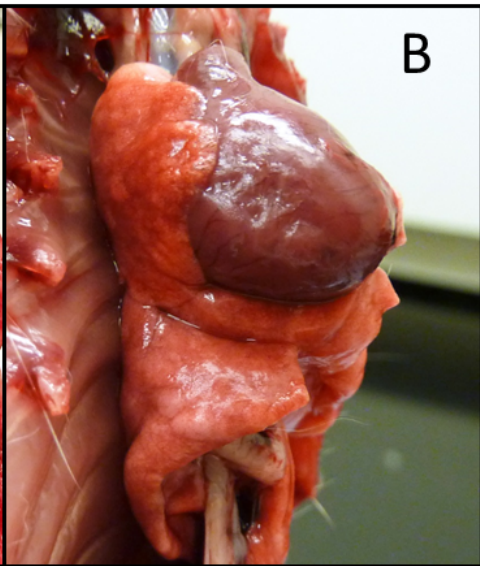

Supplement: Figure S2 [file sph006162212sf2.pdf]

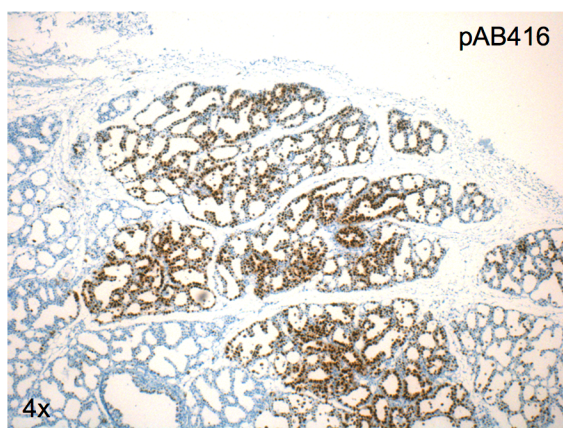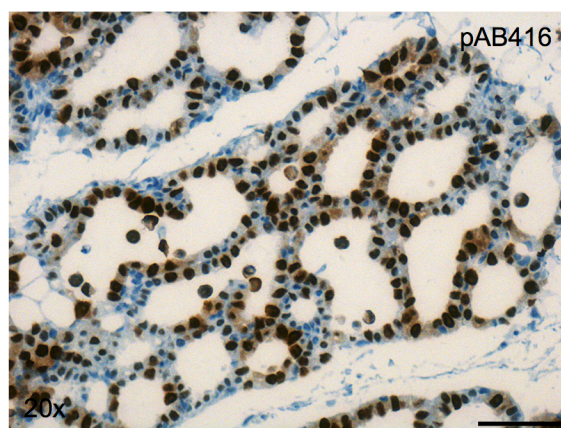

Supplement: Figure S3 [file sph006162212sf3.pdf]
