# Supplementary material for: Identification and Characterization of Novel Rat Polyomavirus 2 in a Colony of X-SCID Rats by P-PIT assay
Source: mSphere. 2016 Dec 21;1(6):e00334-16. doi: 10.1128/mSphere.00334-16 (PMC5177731; doi:10.1128/mSphere.00334-16)
Supplement: Table S1 [file sph006162212st4.pdf]

## Table S1. Primers used in this study

| Primer Name | Sequence | Purpose | Ref. |
|---|---|---|---|
| VP1-1F | CCAGACCCAACTARRAATGARAA | VP1 Consensus PCR | (1) |
| VP1-1R | AACAAGAGACACAAATNTTTCCNCC | VP1 Consensus PCR | (1) |
| VP1-2F | ATGAAAATGGGGTTGGCCCNCTNTGYAARG | VP1 Consensus PCR | (1) |
| VP1-2R | CCCTCATAAACCCGAACYTCYTCHACYTG | VP1 Consensus PCR | (1) |
| HPyV LT-F | ARTWTCAYCCTGATAAAGG | T antigen Consensus PCR | |
| HPyVLT-R | AGCANTCRWAGCAVYAGTA | T antigen Consensus PCR | |
| Rat PyV F2 | GCATATTACCCCAGGCAGAC | Overlapping contigs | |
| Rat PyV R3 | GGTAGTCCCTGCTTGCACTC | Overlapping contigs | |
| Rat PyV F4 | TTCTGCTGCTGCTCAGACTG | Overlapping contigs | |
| Rat PyV R5 | AATTGTCCCTCCATCAGGAC | Overlapping contigs | |
| Rat PyV F6 | CCCTCAGATGGATTTAACGC | Overlapping contigs | |
| Rat PyV F7 | ATGGCGACTATCATGTTTGC | Overlapping contigs | |
| Rat PyV R7 | CCATTCTCATCCAACAGAGGAA | Overlapping contigs | |
| Rat PyV F8 | GTAGAATAGTTGGAGGTGCTGC | Overlapping contigs | |
| Rat PyV R10 | GACAATGATTTCCAAGCAGAAG | Overlapping contigs | |
| Rat PyV F11 | CATCTTCCTGCCGAGTTACC | Overlapping contigs | |
| Rat PyV R12 | ACCTAAAGACCCTATGAGCCTC | Overlapping contigs | |
| Rat PyV F13 | AGCTAACATATAGGTGGATAAGGTG | Overlapping contigs | |
| Rat PyV R1 | CTTCTCCAAAAATGGATCGC | Overlapping contigs | |
| Rat PyV F14 | TGAAATATCAGATCAGTGTTCTGA | Sequencing primers | |
| Rat PyV R14 | ATCCTGTAACACTCCCAAACAACC | Sequencing primers | |
| Beta-actin F | CACACTGTGCCCATCTATGAGG | House-keeping gene PCR | |
| Beta-actin R | TCGAAGTCTAGGGCGACATAGC | House-keeping gene PCR | |

## REFERENCE

1. Johne R, Enderlein D, Nieper H, Müller H. Novel polyomavirus detected in the feces of a chimpanzee by nested broad-spectrum PCR. *J Virol.* 2005 Mar;79(6):3883-7.
