# Supplementary material for: Identification and Characterization of Novel Rat Polyomavirus 2 in a Colony of X-SCID Rats by P-PIT assay
Source: mSphere. 2016 Dec 21;1(6):e00334-16. doi: 10.1128/mSphere.00334-16 (PMC5177731; doi:10.1128/mSphere.00334-16)
Supplement: Table S2 [file sph006162212st5.pdf]

Table S2. Gross findings present in Rat PyV2-infected rats

Organ	Lesion	Size	Severity	Distribution
Lung	Red foci	1-2 mm	Mild	Multifocal
	White foci	1-2 mm	Moderate to severe	Multifocal to coalescing
	Atelectasis	Varies	Mild	Cranioventral
Nasal cavity	Rostral tan-brown crusting	N/A	Mild	Rostral, diffuse

N/A: Not applicable; organ not examined in this group
