# Supplementary material for: Identification and Characterization of Novel Rat Polyomavirus 2 in a Colony of X-SCID Rats by P-PIT assay
Source: mSphere. 2016 Dec 21;1(6):e00334-16. doi: 10.1128/mSphere.00334-16 (PMC5177731; doi:10.1128/mSphere.00334-16)
Supplement: Table S3 [file sph006162212st6.pdf]

Table S3. Histopathologic lesions in Rat PyV2 infected rats

**Organ**	**Lesion**	**Severity by Group**		
		**Adults**	**Weanlings**	**Co-infected with *P. carinii***
Lungs**	Bronchiolar hyperplasia	Moderate	Moderate	Moderate
	Interstitial pneumonia*	Moderate	Severe	Moderate
	Alveolar edema	Mild	Severe	Mild
	Alveolar hemorrhage	Moderate	Mild	Moderate
	Alveolar neutrophils	—	Moderate	—
	Subpleural foamy macrophages	Moderate	—	Moderate
	Bronchial neutrophils and mucous	Mild	—	Severe
Nasal cavity**	Epithelial necrosis	Mild	Severe	N/A
	Neutrophilic and histiocytic infiltrates	Mild	Severe	N/A
	Lymphoplasmacytic and mastocytic infiltrates	Moderate	—	N/A
Harderian gland**	Epithelial necrosis	Severe	—	N/A
	Epithelial hyperplasia	Severe	Mild	N/A
	Glandular atrophy and loss	Severe	—	N/A
	Fibrosis	Moderate	—	N/A
	Mixed inflammatory infiltrate	Moderate	—	N/A
Salivary glands ** (Parotid and Submandibular)	Epithelial necrosis	Severe	Mild	N/A
	Glandular atrophy and loss	Severe	Mild	N/A
	Fibrosis	Severe	Mild	N/A
	Mixed inflammatory infiltrate	Moderate	Mild	N/A
Prostate**	Hyperplasia and dysplasia	Severe	N/A	N/A
Uterus**	Epithelial hyperplasia	Mild	—	N/A
Kidney	Tubular necrosis	Moderate***	—	N/A

*Interstitial pneumonia was characterized by mixed interstitial inflammatory infiltrates and type II pneumocyte hyperplasia

** Intranuclear inclusion bodies were present in epithelial cells of these organs in every group where tissue was examined

***Lesion only present in a single male examined

N/A: Not applicable; organ not examined in this group

Dash (—): Lesion not present
